# Screening for Alcohol Problems: What Makes a Test Effective?

**Published:** 2004

**Authors:** Scott H. Stewart, Gerard J. Connors

**Affiliations:** Scott H. Stewart, M.D., is an assistant professor in the Department of Medicine, School of Medicine and Biomedical Sciences at the State University of New York at Buffalo, Buffalo, New York. Gerard J. Connors, Ph.D., is director and a senior research scientist at the Research Institute on Addictions, State University of New York at Buffalo, Buffalo, New York

**Keywords:** AOD (alcohol and other drug) use screening method, identification and screening for AODD (alcohol and other drug disorders), risk assessment, specificity of measurement, sensitivity of measurement, predictive validity, Alcohol Use Disorders Identification Test (AUDIT)

## Abstract

Screening tests are useful in a variety of settings and contexts, but not all disorders are amenable to screening. Alcohol use disorders (AUDs) and other drinking problems are a major cause of morbidity and mortality and are prevalent in the population; effective treatments are available, and patient outcome can be improved by early detection and intervention. Therefore, the use of screening tests to identify people with or at risk for AUDs can be beneficial. The characteristics of screening tests that influence their usefulness in clinical settings include their validity, sensitivity, and specificity. Appropriately conducted screening tests can help clinicians better predict the probability that individual patients do or do not have a given disorder. This is accomplished by qualitatively or quantitatively estimating variables such as positive and negative predictive values of screening in a population, and by determining the probability that a given person has a certain disorder based on his or her screening results.

The term “screening” refers to the application of a test to members of a population (e.g., all patients in a physician’s practice) to estimate their probability of having a specific disorder, such as an alcohol use disorder (AUD) (i.e., alcohol abuse or alcohol dependence). (For a definition of AUDs and other alcohol-related diagnoses, see the sidebar “Definitions of Alcohol-Related Disorders.”) Screening is not the same as diagnostic testing, which serves to establish a *definite* diagnosis of a disorder; screening is used to identify people who are *likely* to have the disorder. These people are often advised to undergo more detailed diagnostic testing to definitively confirm whether or not they have the disorder. When a screening test indicates that a patient may have an AUD or other drinking problem, the clinician might initiate a brief intervention and arrange for clinical followup, which would include a more extensive diagnostic evaluation (Babor and Higgins-Biddle 2001).

**Definitions of Alcohol-Related Disorders**

A variety of terms are used in the scientific literature to describe alcohol use disorders (AUDs) and other conditions characterized by excessive alcohol consumption. AUDs are disorders for which specific diagnostic criteria exist, as defined in two disease classification systems—the Diagnostic and Statistical Manual of Mental Disorders (DSM), devised by the American Psychiatric Association (APA), and the International Classification of Diseases (ICD), by the World Health Organization (WHO).

***DSM Criteria***

The most recent version of the DSM, the DSM–IV–TR (APA 2000), includes two AUDs, alcohol abuse and alcohol dependence, which have the following diagnostic criteria:

***Alcohol Abuse***. Alcohol abuse is defined as a maladaptive pattern of alcohol use leading to clinically significant impairment or distress, as manifested by the occurrence of one (or more) of the following within a 12-month period:

- Recurrent alcohol use resulting in a failure to fulfill major role obligations at work, school, or home (e.g., repeated absences or poor work performance related to alcohol use; alcohol-related absences, suspensions, or expulsions from school; neglect of children or household).
- Recurrent alcohol use in situations in which it is physically hazardous (e.g., driving an automobile or operating a machine when impaired by alcohol).
- Recurrent alcohol-related legal problems (e.g., arrests for alcohol-related disorderly conduct).
- Continued alcohol use despite having persistent or recurrent social or interpersonal problems caused or exacerbated by the effects of alcohol (e.g., arguments with spouse about intoxication, physical fights).

In addition, the patient must have never met the criteria for alcohol dependence in the past.

***Alcohol Dependence***. Alcohol dependence is defined as a maladaptive pattern of alcohol use leading to clinically significant impairment or distress, as manifested by the occurrence of three (or more) of the following at any time in the same 12-month period:

- Tolerance, as defined by either of the following:
  – A need for increased amounts of alcohol to achieve intoxication or the desired effect.
  – Markedly diminished effect with continued use of the same amount of alcohol.
- Withdrawal, as manifested by either of the following:
  – The characteristic withdrawal syndrome.
  – Use of alcohol to relieve or avoid withdrawal symptoms.
- Drinking alcohol often in larger amounts or over a longer period than was intended.
- A persistent desire or unsuccessful efforts to cut down or control alcohol use.
- A great deal of time spent in activities necessary to obtain alcohol, use it, or recover from its effects.
- Giving up or reducing important social, occupational, or recreational activities because of alcohol use.
- Continued alcohol use despite having a persistent or recurrent physical or psychological problem that is likely to have been caused or exacerbated by alcohol (e.g., continued drinking despite recognition that an ulcer was made worse by alcohol consumption).

Alcohol dependence may include physiological dependence if there is evidence of tolerance or withdrawal. If neither of these is present, alcohol dependence is classified as being without physiological dependence.

***ICD Criteria***

The most recent version of the ICD, ICD–10 (World Health Organization 1993), distinguishes between harmful use and alcohol dependence syndrome. Harmful use is defined as a pattern of alcohol use that is causing damage to health. The damage may be physical (e.g., hepatitis following long-term alcohol use) or mental (e.g., depressive episodes secondary to heavy alcohol intake). Harmful use commonly, but not invariably, has adverse social consequences; social consequences in themselves, however, are not sufficient to justify a diagnosis of harmful use.

The ICD criteria for alcohol dependence syndrome are very similar to those for alcohol dependence in the DSM–IV–TR. They specify that three or more of the following manifestations should have occurred together for at least 1 month or, if persisting for periods of less than 1 month, should have occurred together repeatedly within a 12-month period:

- A strong desire or sense of compulsion to consume alcohol.
- Impaired capacity to control drinking in terms of its onset, termination, or levels of use, as evidenced by either of the following:
  – Alcohol often taken in larger amounts or over a longer period than intended.
  – A persistent desire or unsuccessful efforts to reduce or control alcohol use.
- A physiological withdrawal state when alcohol is reduced or ceased, as evidenced by either of the following:
  – The characteristic withdrawal syndrome for alcohol.
  – Use of the same (or closely related) substance with the intention of relieving or avoiding withdrawal symptoms.
- Evidence of tolerance to the effects of alcohol, such that one of the following occurs:
  – A need for significantly increased amounts of alcohol to achieve intoxication or the desired effect.
  – A markedly diminished effect with continued use of the same amount of alcohol.
- Preoccupation with alcohol, as manifested by one of the following:
  – Giving up or reducing important alternative pleasures or interests because of drinking.
  – Spending a great deal of time in activities necessary to obtain or consume alcohol, or to recover from its effects.
- Persistent alcohol use despite clear evidence of harmful consequences, as evidenced by continued use when the person is actually aware, or may be expected to be aware, of the nature and extent of harm.

In addition to the diagnosis of alcohol dependence, the World Health Organization also uses the term “hazardous use,” which describes a pattern of substance use that increases the risk of harmful consequences for the user. These may include not only physical and mental health consequences but also social consequences. In contrast to harmful use, hazardous use refers to patterns of use that are of public health significance but do not meet the criteria for a current disorder in the drinker. However, the term is not a diagnostic term in the ICD–10.

***Other Terms Used***

In addition to these specific diagnostic terms, various other terms are used in the literature, such as problem drinking, at-risk drinking, and problematic drinking. These terms can differ in their meanings and generally are defined in the context of the specific study.

—Scott H. Stewart and Gerard J. Connors

ReferencesAmerican Psychiatric Association (APA)Diagnostic and Statistical Manual of Mental Disorders. Fourth Edition, Text RevisionWashington, DCAPA2000World Health Organization (WHO)International Statistical Classification of Diseases and Related Health Problems. Tenth RevisionGeneva, SwitzerlandWHO19933376487

Regardless of the context in which screening tests are administered and the subsequent responses, it is important to have an appreciation of the strengths and limitations of screening tests. Accordingly, the main purpose of this article is to review the characteristics of screening tests that influence their usefulness in clinical settings. This includes their validity, sensitivity, and specificity. In addition, the article discusses methods to quantify the likelihood that a patient with a given screening result actually has the disorder (i.e., the postscreen probability). A review of different screening tests, particularly those that can be used in specific settings or with special populations, is beyond the scope of this article. The accompanying table summarizes the features of some of the most commonly used screening instruments. Additional screening tools and their characteristics have been reviewed by Connors and Volk (2003) and are described in the other articles in this issue and the companion issue of *Alcohol Research & Health*.

## What Disorders are Amenable to Screening?

Not all disorders are suitable for screening; in fact, for certain disorders, screening tests may not be helpful or desirable. The main goal of screening is to identify patients at risk for a given disorder or at early stages of the disorder, so that they can begin to receive effective treatment and avoid or ameliorate the morbidity and mortality associated with the disorder. Consequently, disorders should have the following characteristics to be considered suitable for screening:

- They should be a cause of substantial morbidity or mortality.
- Effective treatment should be available that leads to a measurable improvement in morbidity and mortality compared with no treatment.
- Early treatment initiated after a positive screening result should lead to a better outcome than treatment which is initiated later in the disease process, when the disease has produced obvious symptoms that have led to a diagnosis. For example, in a general medical setting, patients should have better outcomes if an intervention is initiated after a screening test, such as the Alcohol Use Disorders Identification Test (AUDIT) (Babor et al. 2001), suggests a pattern of “harmful drinking” than if a diagnosis is made and intervention started after the patient already has developed a more severe condition, such as alcoholic liver disease.
- The disorder should be relatively common because, all else being equal, screening for prevalent disorders is more cost-effective than screening for rare disorders.

AUDs and other drinking problems generally fit these criteria. They are a major cause of morbidity and mortality (NIAAA 2000), are prevalent in the population (NIAAA 2003), and effective treatments are available (Saitz 2005). In addition, because AUDs may have an acute presentation (e.g., alcohol-related trauma or gastrointestinal bleeding) or result in long-term adverse consequences (e.g., liver disease) patients benefit from early detection and intervention. Finally, many people with AUDs never are diagnosed correctly. The next sections therefore will explore the characteristics screening tests must possess in order to be useful and effective.

## Characteristics of Screening Tests Affecting Their Usefulness

Screening tests are designed to be used with members of large populations who have no obvious signs of a particular disease or disorder. For detecting AUDs and other alcohol-related problems, screening may involve the use of biological markers (e.g., liver tests or measurement of a compound called carbohydrate-deficient transferrin) (see Allen et al. 2003) or self-report questionnaires (e.g., the AUDIT, CAGE, and others).

**Table:** Common Alcohol Screening Instruments in Medical Settings*

Measure	Population to Be Screened	Number of Items (Subscales)	Time to Administer (Minutes)
Alcohol Use Disorders Identification Test (AUDIT)	Adults	10 (3)	2
CAGE Questionnaire	Adults and adolescents > 16 years	4	<1
Michigan Alcoholism Screening Test (MAST)	Adults and adolescents	25	8
Self-Administered Alcoholism Screening Test (SAAST)	Adults	35 (2)	5

* Briefer versions of some of these screening instruments (e.g., the MAST and SAAST) also have been tested.

SOURCE: National Institute on Alcohol Abuse and Alcoholism (NIAAA). *Assessing Alcohol Problems: A Guide for Clinicians and Researchers*, 2d ed. NIH Pub. No. 03–3745. Washington, DC: U.S. Dept. of Health and Human Services, Public Health Service, 2003.

Because screening large numbers of people comes at a cost, the screening test should be considered beneficial from the perspective of the society in which it is applied. This means that the test either saves more resources than it utilizes or that the benefits resulting from the screen are perceived to outweigh the cost. Cost-effectiveness is thus determined by factors such as the disease characteristics discussed above, the direct costs of the screening test, the safety of the test, and the validity of the screening test. Validity refers to the screening test’s ability to distinguish those at greater risk for a disorder from those at lower risk. In the development of screening tests, validity is quantified by comparing screening results with a gold standard for diagnosis.

### Validity and the Gold Standard

**Figure 1:**
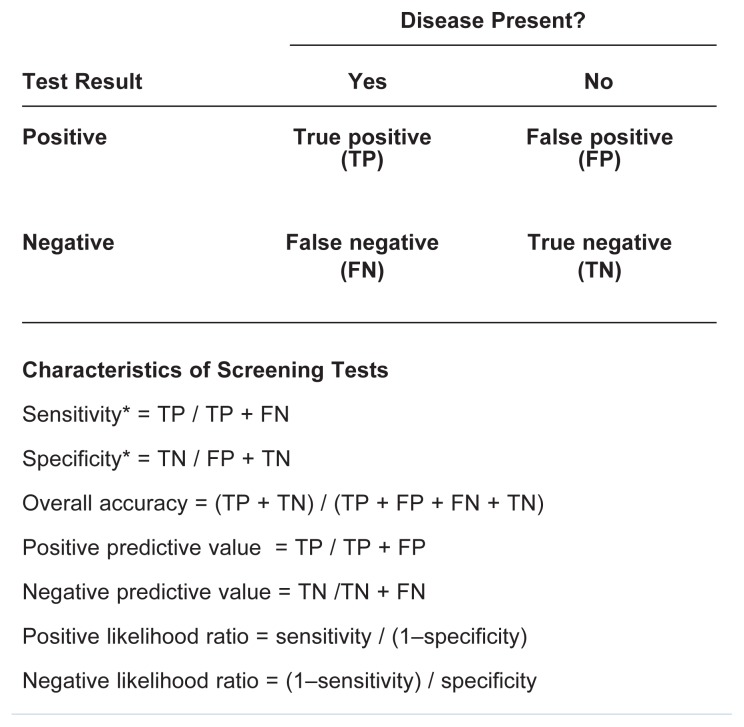
Definitions of terms used to describe characteristics of screening tests.** *Sensitivity and specificity are mathematically expressed as numerical values ranging between 0 and 1. **This illustration assumes that a test can only yield positive or negative results.

A gold standard is a measure that (ideally) correctly identifies every person with the disorder as well as all people without the disorder. Such a test typically is too time consuming or expensive to use for mass screening, but it is perfect for establishing a definitive diagnosis and for judging the validity of screening tests. During this validation process, a group of people with and without a specific disorder complete a screening test and undergo testing using the gold standard. Assuming the gold standard always makes the correct diagnosis, respondents then can be classified into four groups (see figure 1):

- *True positives:* People who have a positive screening result and who have the disorder according to the gold standard test.
- *False positives:* People who have a positive screening result but do not have the disorder according to the gold standard.
- *True negatives:* People who have a negative screening result and do not have the disorder according to the gold standard.
- *False negatives:* People who have a negative screening result but who actually have the disorder according to the gold standard.

An ideal screening test would provide only true positive and true negative results—that is, it would be as accurate as the gold standard for diagnosis. However, screening tests rarely if ever are perfect. In addition, when interpreting the results of screening test evaluations, it is important to keep in mind that often no perfect, or even nearly perfect, gold standard exists. In the case of AUDs, for example, various diagnostic interviews can to some extent lead to different diagnoses (Hasin et al. 1997). This lack of an at least near-perfect gold standard introduces some uncertainty into estimating the validity of screening tests for AUDs.

Specific measures that help assess the usefulness of a screening test are its sensitivity, specificity, and overall accuracy.

### Sensitivity

The term “sensitivity” refers to the ability of a test to correctly identify those people in a population who actually have the disorder. That is, sensitivity represents the probability that a test for a specific disorder will be positive when the disorder truly is present; it ranges in value from 0 to 1 (or equivalently, from 0 percent to 100 percent). The phrase “specific disorder” is important in this context because a screening test can perform differently depending on which disorder or group of disorders is being examined. The AUDIT, for example, will have a different sensitivity when screening for alcohol dependence than when screening for hazardous drinking, and yet another sensitivity when screening for both conditions (Fiellin et al. 2000).

Sensitivity is calculated as the proportion of people with a disease who have a positive screening test. In terms of the four groups of people defined when a screening test is compared with a gold standard, sensitivity is the ratio of true positives over all people who actually have the disorder (that is, true positives plus false negatives) (see figure 1).^1^ A highly sensitive test is desirable when the cost of missing people who actually have a disorder (i.e., who have a false negative screening result) is high. For example, if a screening test is not sensitive enough to correctly identify a commercial airline pilot who exhibits “harmful drinking,” the results (e.g., an intoxicated pilot flying a plane) can lead to potentially catastrophic consequences.

In screening for AUDs, sensitivity can be enhanced by lowering the cutoff scores used to define a positive screening result. For example, the AUDIT consists of 10 questions. Respondents can score between 0 and 4 points on each question, so the total score ranges between 0 and 40 points. (For more information on the AUDIT, see the sidebar “Screening Tests,” on page 28.) Generally, a score of 8 points or higher is considered suggestive of a diagnosis of “hazardous alcohol use.” However, if the cutoff score for hazardous use is lowered to 4 or more points, the sensitivity of the test increases significantly—that is, more people with a drinking problem would have a positive screening result. Such a lowered cutoff score rarely is used, however, because it also would increase the number of false positive results, thereby reducing the test’s specificity, as described in the next section.

### Specificity

Specificity is the test’s ability to identify people in a group who do not have the disorder under investigation. That is, specificity is the probability that a test for a specific disorder will be negative when the disorder is truly absent. Like sensitivity, specificity values range from 0 to 1 (or 0 percent to 100 percent). Specificity is the ratio of people without the disease who screen negative (or true negatives) over all people who actually are without the disease (true negatives plus false positives) (see figure 1). The more specific the test is (i.e., the closer the specificity value is to 1), the fewer people will screen positive for the disease when they do not have it (i.e., the number of false positives approaches 0). A highly specific screening test is desirable when the cost of a false positive result is high. This is less of a problem when screening for drinking problems, because additional testing typically would be performed after a positive screen. Any additional diagnostic evaluations also require additional resources, however, and such resources often are limited.

Screening tests for AUDs can be made more specific by increasing the cutoff point used to define a positive test. For example, when the cutoff value for “hazardous use” in the AUDIT is increased from 8 points to 10 points, a greater proportion of people without a drinking problem will have negative screening results. But because a higher cutoff value also leads to more negative screening results in people who actually meet the diagnostic criteria for hazardous use, raising the cutoff score would simultaneously reduce the test’s sensitivity. Therefore, it is important to balance the sensitivity and specificity of a test, as described below.

### Overall Accuracy

Accuracy is another measure of a screening test’s validity but is less useful than sensitivity and specificity. Accuracy is defined as the proportion of people correctly classified by the test. In other words, it is the ratio of the sum of true positives and true negatives over the entire study population (see figure 1). The usefulness of accuracy in characterizing a test is limited by the fact that it is not an inherent characteristic of the test but varies with the prevalence of a disorder in a population (i.e., the higher the prevalence, the greater the accuracy). In most populations, the prevalence of AUDs is significantly less than 50 percent. With this prevalence rate, overall accuracy is almost equal to specificity and does not provide additional value in estimating the validity of a screening test (Alberg et al. 2004). Therefore, it is preferable to use sensitivity and specificity to determine a test’s validity.

## Balancing Sensitivity and Specificity

**Figure 2:**
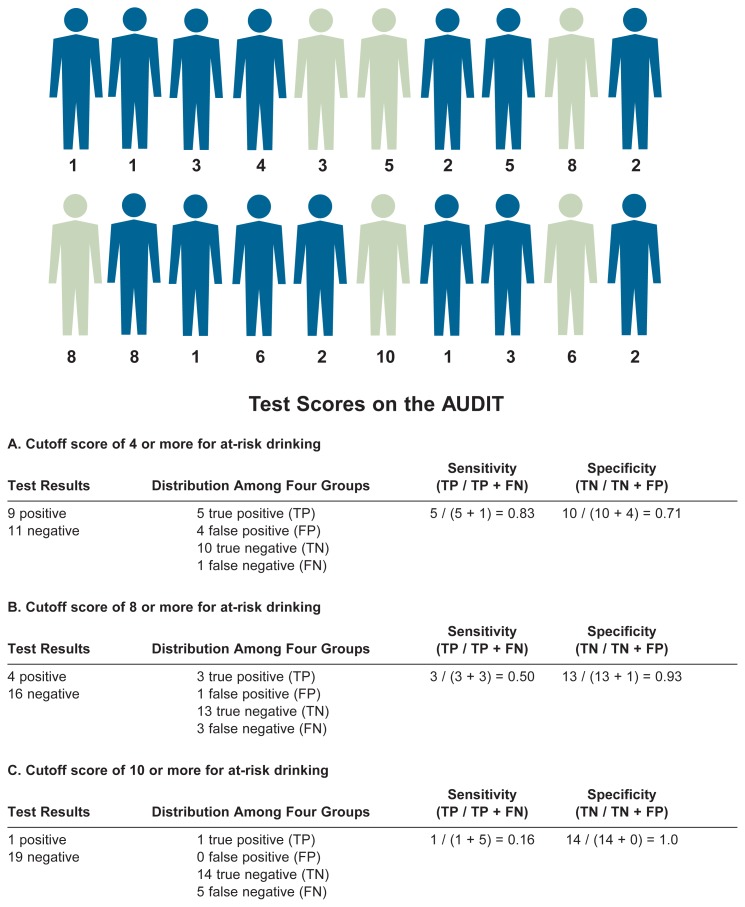
Illustration of the effects of changes in the cutoff score of a screening test (e.g., the AUDIT) on the test’s sensitivity and specificity. Among the 20 hypothetical people screened, 6 meet the gold standard criteria for at-risk drinking (green), and 14 are nonrisk drinkers (blue). Numbers below the hypothetical people indicate their test scores on the AUDIT.

As the discussion in the previous section indicated, for an ideal screening test both sensitivity and specificity would be close to 1, so that most people are classified correctly and only a few would have a misleading test result. In practice, however, this rarely is the case, and striking a balance between sensitivity and specificity is necessary. For example, as mentioned earlier, lowering the cutoff score for a positive test result on the AUDIT from 8 to 4 points can increase the test’s sensitivity—that is, the number of people with a drinking problem classified as having a positive test result would go up. But because the increase in positive tests would include not only people who actually meet the criteria for hazardous alcohol use (i.e., are true positives) but also some who do not meet those criteria (i.e., are false positives), it also would mean a decrease in the test’s specificity (see figure 2).

So how is it possible to choose an appropriate cutoff score for differentiating a positive from a negative result on a screening test? The answer depends on the relative consequences of false positive versus false negative tests—that is, is it more harmful to the individual or to society as a whole if a person is wrongly classified as having a drinking problem, or if the person is wrongly classified as not having a drinking problem?

The trade-off between sensitivity and specificity often is illustrated using a type of graphic called a receiver operator characteristic (ROC) curve (see the sidebar “Receiver Operator Characteristic [ROC] Curves”). ROC curves plot the number of true positives (expressed as the sensitivity of a test) on the *y*-axis against the number of false positives (expressed as 1 minus the specificity of the test) on the *x*-axis at different cutoff scores. The resulting graph can help clinicians and researchers identify the cutoff value with the best possible combination of specificity and sensitivity for a given test. For example, researchers have used an ROC curve to identify an optimal cutoff score for the AUDIT when screening for “at-risk” drinking^2^ in a primary care setting (Volk et al. 1997). When screening for at-risk drinking in this study population, a cutoff score of 4 provided roughly equal sensitivity and specificity (i.e., balanced false positives and false negatives) and maximized accuracy. It is important to note, however, that studies designed to validate the AUDIT in other populations and for other drinking behavior categories typically have selected higher cutoff scores as optimal for their conditions. Accordingly, it is essential to validate screening tests for a *specific* disorder or group of disorders in *populations that are similar* to the populations that will be screened using those tests. Whether a test’s validity has been adequately established for a specific population is often a matter of judgment.

**Receiver Operator Characteristic (ROC) Curves**

A receiver operator characteristic (ROC) curve is a mathematical tool for assessing the usefulness of a screening test at different cutoff scores. To generate an ROC, one needs to know the test’s specificity and sensitivity, both of which are mathematically expressed as numerical values between 0 and 1. ROC curves plot the number of true positives (represented as sensitivity) versus the number of false positives (represented as [1−specificity]). For a test that is no more accurate than chance alone, the values for these two variables at different cutoff scores would fall on the diagonal indicated by the dashed line in the figure. The closer the curve of a screening test follows the left-hand border and then the top border, the more accurate the test is. The ideal cutoff value is the point in the curve that is located closest to the upper left-hand corner.

The figure shows the ROC curve for the Alcohol Use Disorders Identification Test (AUDIT) when used to screen for at-risk drinking in a primary care setting. The numbers along the curve represent the various cutoff points analyzed. Based on these data, the AUDIT had some value at all cutoff scores because all points on the curve were above the diagonal. With low cutoff scores, the AUDIT was highly sensitive (i.e., minimized the number of false negatives) but had *relatively* low specificity. At high cutoff scores, the test was highly specific (i.e., minimized false positives) but had poor sensitivity. Under the conditions assumed in this analysis (i.e., when screening for at-risk drinking in this study population), the best cutoff score was 4 because it provided roughly equal sensitivity and specificity (i.e., balanced false positives and false negatives).

**Figure:**
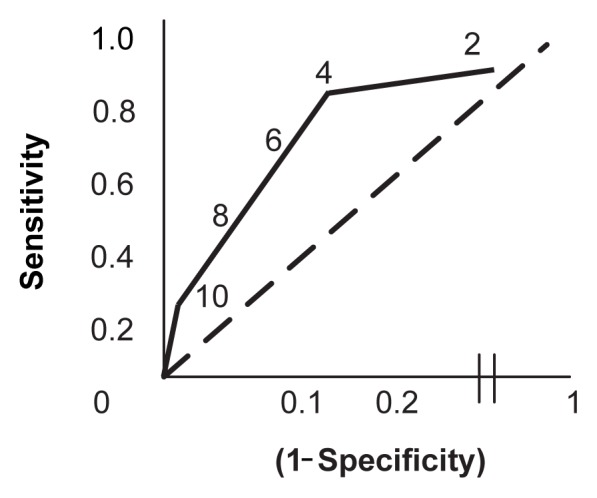
Graph showing the sensitivity and specificity of the AUDIT. Numbers along the curve represent the cutoff points analyzed. SOURCE: Estimated from data by Volk et al. 1997.

—Scott H. Stewart and Gerard J. Connors

ReferenceVolkRJSteinbauerJRCantorSBHolzerCEThe Alcohol Use Disorders Identification Test (AUDIT) as a screen for at-risk drinking in primary care patients of different racial/ethnic backgroundsAddiction92219720619979158231

## Methods to Quantify Postscreen Probability

As described above, all screening tests yield a certain number of false positive and false negative results. Therefore, an important question when evaluating a screening test is: What is the probability that, given a certain test result, the person actually has the disease? This also is known as the postscreen probability. It can be determined using several measures, including the positive and negative predictive values and the positive and negative likelihood ratios. These measures are discussed in the following sections.

### Predictive Values

#### Positive Predictive Value

The positive predictive value (also known as the predictive value of a positive test) is defined as the proportion of patients with positive tests who actually have the disease (see figure 1). Thus, the positive predictive value depends on the ability of the screen to correctly classify people (i.e., identify true positives and true negatives). It also depends on the prevalence of the disorder in the screened population: The higher the prevalence of the disorder that is being screened for, the higher the positive predictive value of the screening test.

This relationship can be illustrated with the following example: When using the AUDIT to screen for at-risk drinking in a population of primary care patients, Volk and colleagues (1997) determined that with a cutoff score of 4 to indicate at-risk drinking, the test’s sensitivity is 85 percent and its specificity is 84 percent compared with a gold standard (i.e., more in-depth diagnostic interviewing). Using these assumptions, the positive predictive value of the AUDIT would be 0.37 if the prevalence of at-risk drinking in the population is 10 percent, but 0.57 if the prevalence of at-risk drinking is 20 percent. (For a detailed description of how positive predictive value is calculated in this example, see the sidebar “Calculating Predictive Values.”) In other words, the probability that a patient with a positive AUDIT screening result actually is an at-risk drinker would be 37 percent if the prevalence of at-risk drinking is 10 percent, and 57 percent if the prevalence of at-risk drinking is 20 percent. Thus, with different prevalences of at-risk drinking, the same test with the same cutoff values has greatly differing predictive values. And as the prevalence of the disorder increases, the positive predictive value also will continue to increase. This does not imply, however, that screening should not be done in populations with a low prevalence of the disease. Instead, this observation highlights the need for additional, more extensive diagnostic testing in people with a positive screening result to ensure that they actually have the disorder. In general, the extent to which a positive screening result indicates that a person has an increased likelihood of actually having the disorder under investigation depends on the prevalence of the disorder and the test’s validity.

**Calculating Predictive Values**

Predictive values indicate the probability that a person with a positive result on a screening test actually has the disorder being screened for (positive predictive value) or that a person with a negative screening result truly does not have the disorder (negative predictive value). The positive predictive value is calculated as the ratio of true positives over true positives plus false positives; the negative predictive value is calculated as the ratio of true negatives over true negatives plus false negatives (for definitions, see figure 1 in the main article).

Both the positive and the negative predictive value depend on the prevalence of the disorder in the population. This can be illustrated with the hypothetical example of using the AUDIT to screen for at-risk drinking in a population of 1,000 primary care patients, assuming two different prevalence rates (10 percent and 20 percent) for at-risk drinking in that population. For such a population, Volk and colleagues (1997) determined a sensitivity of 85 percent and a specificity of 84 percent if the AUDIT was used with a cutoff score of 4.

If the prevalence of at-risk drinking is assumed to be 10 percent—that is, 100 patients actually are at-risk drinkers and 900 patients are nonrisk drinkers—the positive predictive value can be calculated as follows:

- A sensitivity of 85 percent means that 85 of the 100 at-risk drinkers would test positive and therefore would be true positives; the remaining 15 patients would test negative and therefore would be false negatives.
- A specificity of 84 means that 756 of the 900 nonrisk drinkers would test negative and therefore would be true negatives; the remaining 144 patients would test positive and therefore would be false positives.
- As a result, the positive predictive value—the ratio of true positives over true positives plus false positives—would be 85/(85 + 144) = 0.37. Thus, in this example, the probability that a patient with a positive screening result is an at-risk drinker is 37 percent. (In comparison, without a screening test, every person’s probability of being an at-risk drinker would be 10 percent based on the prevalence of at-risk drinking in that population.)

If the prevalence of at-risk drinking is assumed to be 20 percent—that is, 200 patients actually are at-risk drinkers and 800 patients are nonrisk drinkers—the positive predictive value can be calculated as follows:

- With a sensitivity of 85 percent, 170 of the 200 at-risk drinkers would test positive and would be true positives, and 30 patients would test negative and would be false negatives.
- With a specificity of 84 percent, 672 of the 800 nonrisk drinkers would test negative and would be true negatives; the remaining 128 would test positive and would be false positives.
- As a result, the positive predictive value is now 170/(170 + 128) = 0.57. Thus, the probability that a patient with a positive test result really is an at-risk drinker is 57 percent.

Therefore, with the different assumptions regarding the prevalence of at-risk drinking, the AUDIT used with the same cutoff scores has greatly differing positive predictive values.

The same reasoning applies to negative predictive value, except that the relationship between prevalence and predictive value is inverse: the higher the prevalence, the lower the negative predictive value. For example, using the AUDIT example above, the negative predictive value at a prevalence of 10 percent is calculated as 756/(756 + 15) = 0.98. In other words, the likelihood that a person with a negative AUDIT result is an at-risk drinker is 2 percent (compared with an estimate of 10 percent based solely on the prevalence of the disease). If the prevalence of at-risk drinking is assumed to be 20 percent, then the negative predictive value in the AUDIT example is 672/(672 + 30) = 0.96, meaning that the probability that a person with a negative test result is an at-risk drinker is 4 percent. With increasing prevalence, the negative predictive value will continue to deteriorate.

—Scott H. Stewart and Gerard J. Connors

ReferenceVolkRJSteinbauerJRCantorSBHolzerCEThe Alcohol Use Disorders Identification Test (AUDIT) as a screen for at-risk drinking in primary care patients of different racial/ethnic backgroundsAddiction92219720619979158231

#### Negative Predictive Value

Negative predictive value (also known as predictive value of a negative result) is defined as the proportion of patients who test negative and who do not have the disease (true negatives) among all patients with negative test results. Mathematically, it is equal to the ratio of true negatives over true negatives plus false negatives (see figure 1).

Like positive predictive value, negative predictive value depends on the validity of the screening test and the prevalence of the disorder. However, in this case the relationship is inverse: the higher the prevalence of the disorder in the population, the lower the negative predictive value. This means that a negative screening result is less helpful in ruling out the disease if the prevalence of the disease in the population is high. Continuing with the AUDIT example from Volk and colleagues, the postscreen probability for at-risk drinking among patients screening negatively with a cutoff of 4 was 2 percent, given 10 percent prevalence in the population. At a prevalence of 20 percent, the postscreen probability for a negative result was 4 percent. Analogous to the positive predictive values, negative predictive values illustrate that a negative screening result does not necessarily rule out a disorder. The extent to which a negative screening result indicates that a person has a decreased risk of actually having the disorder under investigation depends on the prevalence of the disorder in the population and the test’s validity.

### Limitations of Predictive Values

Positive and negative predictive values are useful when assessing postscreen probabilities for disorders with a known prevalence in the screened population. In these situations, predictive values provide average postscreen probabilities for all members of the screened population with a particular test result. For example, based on a known prevalence for current alcohol dependence in the screened population of 6 percent, a positive screen for dependence may then increase the probability that a person is alcohol dependent from 6 percent to 25 percent. Both the prescreen probability of 6 percent and the postscreen probability of 25 percent, however, represent an average risk for members of the population. Predictive values do not consider a person’s additional risk factors, such as a family history of alcohol dependence, that may modify both the prescreen and postscreen risk for dependence in that person. A method for incorporating individual risk factors in clinical settings is based on likelihood ratios, which are discussed in the next section.

## Likelihood Ratios

In clinical settings, the physician often has additional information on a patient relevant to that patient’s risk for drinking problems. The use of likelihood ratios allows the clinician to incorporate a specific patient’s prescreen risk for a drinking problem into estimating postscreen probabilities. A likelihood ratio is the ratio of two probabilities—the probability of a given test result among people with the disease divided by the probability of that test result among people without the disease. For example, a likelihood ratio for at-risk drinking would be the probability that an at-risk drinker has a certain test result on the AUDIT divided by the probability that a nonrisk drinker has that result on the AUDIT. Depending on whether one assesses patients with positive test results or negative test results, the resulting likelihood ratios are known as positive likelihood ratio and negative likelihood ratio. The following sections discuss the clinical use of likelihood ratios because they are frequently presented as characteristics of screening tests. In actual practice, however, the results of screening tests applied to individual patients who are not already clinically suspected of having a drinking problem are interpreted dichotomously (i.e., positive or negative). A positive result will lead to additional diagnostic evaluation, and a negative result will preclude further evaluation.

### Positive Likelihood Ratio

The positive likelihood ratio in the AUDIT example used earlier (Volk et al. 1997) is the probability that an at-risk drinker has a positive test result divided by the probability that a nonrisk drinker has a positive test result. It represents the ratio of true positives to false positives. Mathematically, it is calculated as the ratio of sensitivity over [1−specificity] (see figure 1). In the AUDIT example with a cutoff of 4 (i.e., with a sensitivity of 85 percent and a specificity of 84 percent), the positive likelihood ratio would be calculated as 0.85/[1 − 0.84] = 5.3. Thus, the positive likelihood ratio (like the negative likelihood ratio) is a factor that is inherent in a given test—if one knows the sensitivity and specificity of a test, one can calculate the test’s likelihood ratios.

This positive likelihood ratio, together with information on other risk factors for at-risk drinking in a given patient, can be used to calculate that patient’s odds or probability^3^ of being an at-risk drinker. To illustrate this process, imagine the following example: A primary care physician has two 40-year-old male patients who are being treated for high blood pressure. Patient 1 was divorced about 1 year ago, seems depressed, and has poorly controlled blood pressure and slightly abnormal levels of certain liver enzymes. Based only on his history, the physician estimates this patient’s probability of being an at-risk drinker to be 40 percent. Patient 2 appears well and has excellent blood pressure control. The physician estimates his probability of being an at-risk drinker to be 20 percent (equal to the prevalence of at-risk drinking in the local population). Both of these patients have AUDIT results above the cutoff score of 4 chosen by the physician. Through some mathematical calculations based on the estimates of the patients’ individual probabilities of being at-risk drinkers and the AUDIT’s positive likelihood ratio of 5.3 (when using a cutoff score of 4), the physician estimates the post-test probability of Patient 1 being an at-risk drinker to be 0.78 (or 78 percent). In contrast, the post-test probability of Patient 2 is calculated to be 0.56 (or 56 percent).^4^

This example illustrates how a clinician can estimate a specific patient’s probability for being an at-risk drinker following a positive screening test. Similar calculations can be performed based on negative screening results, as described in the next section.

### Negative Likelihood Ratio

The negative likelihood ratio is the probability that a person with a disorder, such as at-risk drinking, has a negative test result (e.g., on the AUDIT) divided by the probability that a person without the disorder has a negative test result. It represents the ratio of false negatives to true negatives and is calculated as the ratio of [1−sensitivity] over specificity (see figure 1). For example, for the AUDIT with a cutoff score of 4, the negative likelihood ratio is [1 − 0.85]/0.84 = 0.18.

Analogous to the positive likelihood ratio, the negative likelihood ratio can be used to calculate a specific patient’s probability of having a disease (e.g., being an at-risk drinker) based on the patient’s history and a negative result on the screening test. For instance, assuming that the two patients in the previous situation both had negative screening results on the AUDIT, calculations to determine the postscreen probability of at-risk drinking would yield values of 11 percent for Patient 1 and 5 percent for Patient 2.^5^

This example demonstrates that likelihood ratios can be useful for predicting the risk in individual patients for a certain disorder; however, there are some limitations to their use. For example, a clinician must have good clinical acumen to accurately predict a given patient’s probability of having the disorder based on the patient’s history (i.e., the patient’s pretest probability), which is needed to estimate the post-test probability of having the disorder as accurately as possible.

## Summary

Screening for AUDs and other drinking problems is warranted in a variety of settings and contexts because these conditions have a relatively high prevalence, can lead to substantial adverse consequences for individuals and society, and can be significantly improved by appropriate treatment. Screening tests should be validated in populations similar to the one being tested and for the specific disorder or group of disorders of interest. The selection of appropriate cutoff scores that balance sensitivity and specificity is a key consideration when using screening tests. With the help of appropriately conducted screening tests, clinicians can better predict the probability that individual patients do or do not have a given disorder. Specific examples of screening tests for AUDs and other alcohol-related problems, as well as of subsequent brief interventions, are highlighted in the remaining articles in this and the companion issue of *Alcohol Research & Health*.

## Footnotes

1 This definition implies that as sensitivity approaches 1, the probability of a false negative result approaches 0.

2 “At-risk” drinking in that study was defined as any pattern of use or alcohol-related consequences that ruled out nonproblem drinking (e.g., drinking in excess of national guidelines, meeting the criteria for hazardous and harmful use, or meeting the criteria for abuse and dependence).

3 Note that “odds” and “probability” are not the same. In mathematical terms, odds = probability/[1−probability], and probability = odds/[odds + 1].

4 Note that Patient 2 has the same post-test probability that was estimated using the positive predictive value (accounting for rounding error), because he was assigned a pretest probability equal to the population prevalence.

5 Again, Patient 2, with an estimated pretest probability equal to the population prevalence, has the same post-test estimate as that obtained with the negative predictive value approach (after accounting for rounding error).
